# Invasive paper wasp turns urban pollinator gardens into ecological traps for monarch butterfly larvae

**DOI:** 10.1038/s41598-020-66621-6

**Published:** 2020-06-12

**Authors:** Adam M. Baker, Daniel A. Potter

**Affiliations:** Department of Entomology; S-225 Agriculture Science Bldg. N., University of Kentucky, Lexington, KY 40546-0091 USA

**Keywords:** Ecology, Zoology

## Abstract

Invasive species can be particularly disruptive when they intersect with organisms of conservation concern. Stabilizing the declining eastern migratory population of monarch butterflies (*Danaus plexippus*) is projected to require extensive habitat restoration across multiple land use sectors including metropolitan areas. Numerous conservation programs encourage urban citizens to plant gardens with milkweeds, the obligate larval host plants of the monarch. Here, we show that predation by *Polistes dominula*, an invasive paper wasp that is particularly abundant in urban settings, can turn such sites into ecological traps for monarch larvae. *Polistes dominula* was the predominant paper wasp seen foraging in central Kentucky pollinator gardens. In 120 observed encounters with monarch larvae on milkweeds in gardens, most second to fourth instars were killed, whereas most fifth instars escaped by thrashing or dropping. The wasps bit and carried off second instars whole, whereas third and fourth instar kills were first gutted, then processed and carried away piecemeal. Predation on sentinel larvae was much higher in urban gardens than in rural settings. The wasps exploited ornamental butterfly “hibernation boxes” in pollinator gardens as nesting habitat. *Polistes dominula* is an under-recognized predator that may diminish the urban sector’s contributions to monarch habitat restoration.

## Introduction

Invasive species can be particularly disruptive when they intersect with organisms of conservation concern^1^. Urban ecological restoration can sometimes create ecological traps by attracting native species to colonize patches of semi-natural habitat where they incur inordinately high mortality from exotic natural enemies^2,3^. For example, songbirds drawn to naturalized suburban habitat for nesting may suffer heavy predation by (non-native) domestic cats^4,5^. Urbanization itself can magnify such interactions by providing nesting sites or other resources for synanthropic invasive predators^6–8^. As urban citizens increasingly plant gardens to support native pollinators and other biodiversity^3,9^, it is important those efforts do not inadvertently create ecological traps for species they are intended to benefit.

Populations of the monarch (*Danaus plexippus*), an iconic migratory North American butterfly, are declining^10,11^. To help offset this decline, conservationists are encouraging planting milkweeds (*Asclepias* spp.), the monarch’s obligate larval host plants, across the breeding range^12^. Despite the public’s enthusiasm for monarch-friendly gardening^13,14^, and projections that restoring enough milkweed to ensure a stable monarch population will require participation by the urban sector^12,15^, the conservation value of urban milkweed gardens remains uncertain. Such gardens attract ovipositing adults, often with higher egg-loading per plant than occurs in natural milkweed stands^16–20^, but they could also be ecological traps if they expose monarchs to increased risk of predation, disease, or abiotic mortality factors.

*Polistes dominula*, the so-called European paper wasp, was first reported in North America in the 1970s where it has since become widespread^21,22^. Its strong proclivity to nest in sheltered places associated with buildings and other man-made structures contributes to its invasion success in urban settings^23^. In addition, its strategy of forming nests with multiple, often unrelated, foundresses results in high nest survival and a competitive edge over sympatric native *Polistes* species^21,22^. Paper wasps find arthropod prey by hovering or walking on plants^24,25^. Prey are killed by biting, masticated to manageable size, flown to the nest either whole or piecemeal, and fed to the wasps’ developing larvae^24,25^. Although *P. dominula* are opportunistic, generalist predators, individuals often return to hunt at sites of previous success^24^. Although the wasps do not actively recruit nest mates, they are attracted to other individuals’ processing of prey^25^, authors’ observations.

While conducting research in urban pollinator gardens^18,19^ we observed *P. dominula* attacking monarch larvae. Paper wasp predation has not previously been studied in the context of butterfly gardens, but given *P. dominula*’s synathropy^22^, we hypothesized it may pose particular danger to monarch larvae in urban settings. Here, we show that *P. dominula* is the predominant paper wasp foraging in urban gardens in central Kentucky, document higher *Polistes* predation on monarchs in urban gardens compared to more rural settings, and describe the behavior and fate of monarchs attacked by *P. dominula* in gardens. We also show that so-called butterfly “hibernation boxes”^26^ may be exploited by *P. dominula* as nesting habitat. Our findings highlight *P. dominula* as an under-recognized predator that could potentially diminish the urban sector’s contributions to monarch conservation.

## Results

### Assessing *P. dominula* prevalence in urban gardens

**Figure 1:**
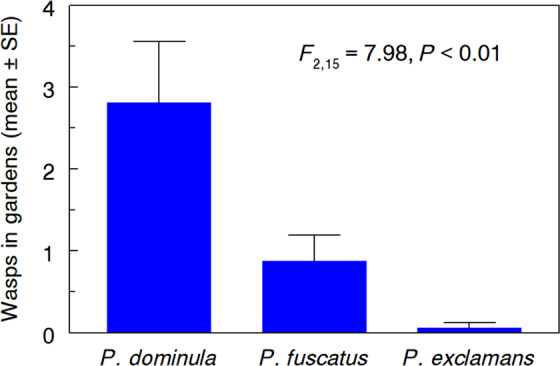
Prevalence of *Polistes dominula*, *P. fuscatus*, *and P. exclamans* foragers based on 60 min observations in each of 16 urban pollinator gardens.

*Polistes dominula* foragers (n = 45) were observed in 10 of the 16 urban pollinator gardens surveyed for paper wasps during July. Two native paper wasp species, *Polistes fuscatus* (n = 14) and *Polistes exclamans* (n = 1) were also observed in some gardens, but in fewer numbers overall (F_2,15_ = 7.98, *P* = < 0.01; Fig. 1). No wasps were observed in three of the 16 gardens, and in three others only *P. fuscatus* was seen.

### *Polistes dominula* encounters with monarch larvae in gardens

**Figure 2:**
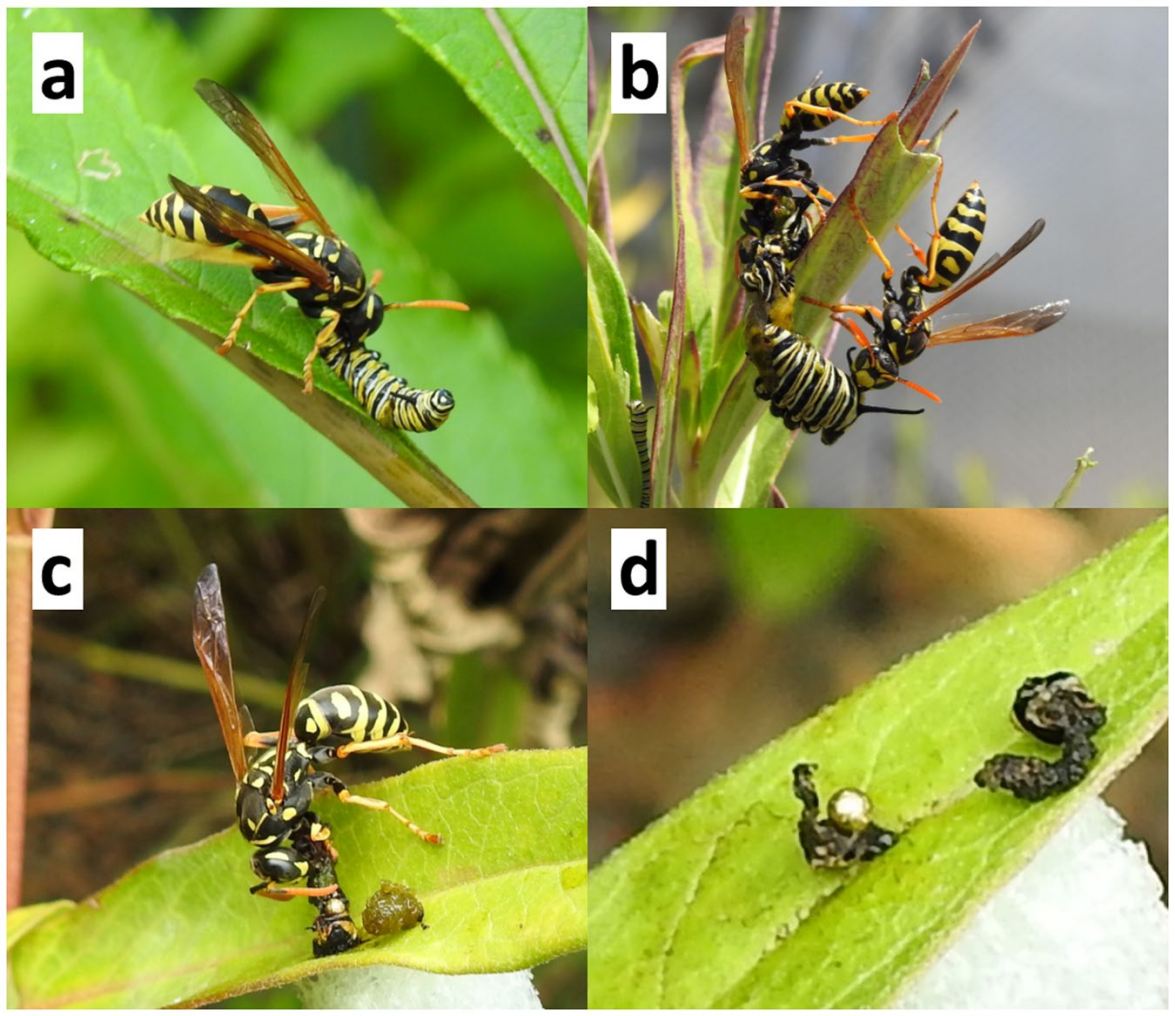
*Polistes dominula* predation on monarch larvae: (**a**) attack on free-feeding 2nd instar, (**b**) second wasp attracted to another’s kill of free-feeding fifth instar, (**c**) wasp gutting pinned sentinel third instar, (**d**) excised gut and head capsule indicative of *P. dominula* attack on third instar.

*Polistes dominula* attacked second to fifth instar monarch larvae placed on swamp milkweed in urban pollinator gardens (Fig. 2a,b; Table 1). Relative proportions preyed upon or escaping such encounters differed among instars, as did the behavior of wasps and larvae (Table 1). Second to fourth instars were more likely than fifth instars to be killed. Wasps encountering second instars mostly struck, bit, and carried off their prey intact, whereas in nearly all predation on third instars, the wasp first excised the larval gut, which was left on the leaf, and then macerated the remains into a ball and flew off with them. Some second and third instars escaped by dropping, with or without silk, but on one occasion we saw a wasp follow the strand down and carry off the larva. Fourth instars were gutted as above, macerated, and processed into manageable pieces, the wasp often taking multiple trips to carry them back to the nest (Table 1). We observed other wasps trying to steal prey pieces while the original wasp was still processing its kill, or to take pieces left behind. Fourth instars escaping predation either dropped or thrashed in response to the wasp’s attack. Nearly all (28/30) fifth instars escaped, either by violently thrashing or dropping. Both observed fifth instar kills were processed by multiple wasps (Fig. 2b). In >52 h of observation, we saw no predation by natural enemies other than *P. dominula*.

**Table 1:** Outcome of 120 encounters (30 per instar) between *Polistes dominula* and sentinel monarch butterfly larvae feeding on swamp milkweed (*Asclepias incarnata*) plants in urban pollinator gardens.

Instar	Outcome	Total^c^	Wasp kill behaviors (in sequence)^a^				Larval escape behaviors^b^		
			S,C^d^	S,G,C	S,G,P	S,W,G,P	D	DSk	T
2nd	Killed	23	21	2					
	Escaped	7					5	2	
3rd	Killed	24	2	20	2				
	Escaped	6					5	1	
4th	Killed	20		4	13	3			
	Escaped	10					5		5
5th	Killed	2				2			
	Escaped	28					8		20

^a^Wasp behaviors resulting in kill: S = strike, G = gut, C = carry off, W = wait, P = process (cut into pieces, then carry off in multiple trips).

^b^Larval behaviors leading to escape: D = drop, DSk = drop on silk, T = thrash.

^c^Proportion of larvae killed or escaped differs significantly between instars (χ^2^ = 43.5, df = 3, *P *≤ 0.001).

^d^Includes one 2nd and one 3rd instar that dropped on silk, then was found by the wasp and carried off intact.

### Predation on monarch larvae in urban and rural settings

**Figure 3:**
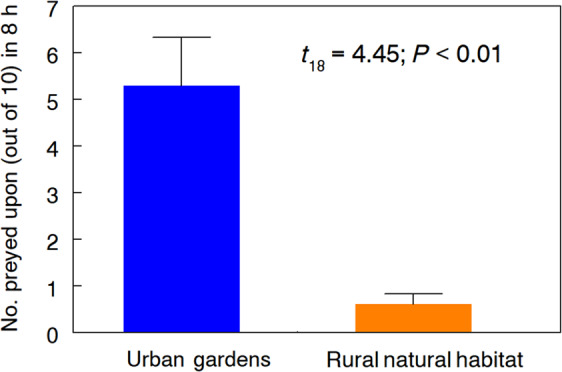
Predation of sentinel third and fourth instar monarch larvae on swamp milkweeds placed in urban pollinator gardens or rural meadow habitat (n = 10 sites of each type). Data are mean (SE) number of larvae (out of 10) per site taken after 8 h of diurnal exposure.

Sentinel third and fourth instar monarch larvae on swamp milkweeds placed in 10 urban pollinator gardens sustained significantly more predation than did cohorts on milkweeds placed in rural meadow habitat bordered by woodlots (Fig. 3). In many cases, the larva’s excised digestive tract was left on the plant near the pin that had secured it (Fig. 2c,d), indicative of predation by *Polistes*. Urban garden sites were close to buildings (mean distance 6.5 ± 1.3 m; range 3–16 m), whereas rural exposure sites were significantly farther away from the nearest building or other structure (257 ± 15 m; range 184–340 m; *t*_18_ = 16.8, *P* < 0.001).

### Wasp exploitation of butterfly boxes in pollinator gardens

**Figure 4:**
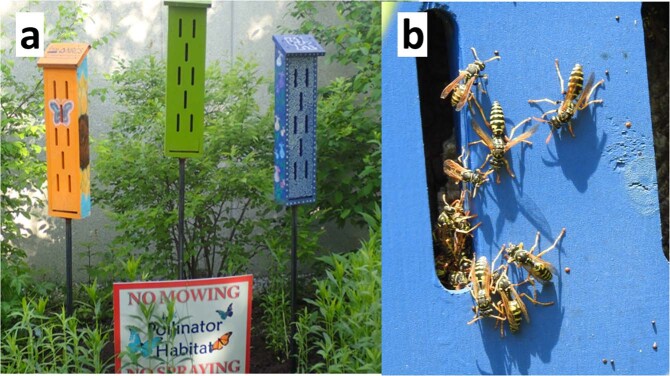
(**a**) Butterfly “hibernation boxes” in urban pollinator gardens; (**b**) Thirteen of 22 boxes in six urban pollinator gardens contained active *P. dominula* nests.

Sixteen of 22 butterfly “hibernation boxes” (Fig. 4a) in six pollinator conservation gardens on University of Kentucky’s campus contained *Polistes* wasp nests. Thirteen boxes were occupied by *P. dominula*, two by *P. fuscatus*, and one by *P. exclamans*. We saw no evidence of butterflies using the boxes, although some did contain spiders or mantis oothecae.

## Discussion

Paper wasps are abundant in most temperate ecosystems where they exert strong selective pressure on lepidopteran larvae^24^. When invasive *Polistes* spp. are introduced to new areas they compete with native species for niche availability^21,22,27^ and may elevate predation pressure, putting prey species at risk of population decline^28^. Since being introduced into the eastern United States in the late 1970s, *P. dominula* has become widely established in North America^29^, especially in urbanized settings where the types of sheltered nesting sites it prefers are plentiful^21,23^. Although *Polistes* spp. can be efficient biocontrol agents for lepidopteran pests of urban agriculture [e.g.^30,31^], this study highlights the potential for *P. dominula*, in particular, to cause substantial mortality of second to fourth instar monarchs in urban butterfly gardens.

Monarchs typically incur high (90–95% or more) mortality from egg to fifth instar^32–36^. Host plant defenses cause some, especially of early instars^37^, but invertebrate natural enemies probably account for more^34^. Monarch larvae are preyed upon by ants, spiders, predatory bugs, mantids, lady beetles, vespid wasps, or other arthropods^32,34,38^ and parasitized by tachinid flies^39^. While numerous studies have inferred causes of predation by tracking stage-specific disappearance of monarch eggs and larvae in the field (e.g.^32–36^], few have observed and quantified predation events directly. In the literature, *P. dominula* has received scant, mainly anecdotal, mention as predator of monarch larvae in field settings.

Besides killing them outright, encounters with *P. dominula* might indirectly reduce fitness of monarch larvae by interrupting their feeding, thus reducing rate of growth, or by causing them to drop from the plant where they might be exposed to ground-dwelling predators. Survivors would then need to invest additional time crawling back up to preferred sites that might otherwise be spent feeding. Such indirect effects of harassment by *Polistes* spp. have been shown to significantly amplify direct impacts of predation in other plant-caterpillar systems^40^. We did not track fate of monarch larvae that escaped encounters with wasps, but such indirect effects warrant further study.

The one previous published study of *P. dominula* predation on monarchs deployed active wasp nests transplanted to a greenhouse to test the hypothesis that monarch larvae raised on different *Asclepias* species present a spectrum of palatability^25^. In it, captive free-flying wasps took monarch larvae regardless of the cardenolide content of the milkweed species upon which they had been reared, although overall, ones that had fed on milkweeds with relatively low cardenolide content were preferred^25^. That study also concluded, based on trials in which small, medium-sized, or large larvae were all presented simultaneously, that second through early third instars are largely ignored, whereas we observed *P. dominula* to quickly find and attack individuals of all sizes in gardens. Notably, larvae reared on *A. incarnata*, *A. syriaca*, and *A. tuberosa*, three species commonly planted in butterfly gardens^18,19^, all were palatable^25^.

When processing lepidopteran prey, *Polistes* spp. may use their mandibles to excise guts that contain plant material from the balled masses of tissue they carry back to their nests^24,25^. Such behavior may be selective, depending on what plant the prey had fed upon^25^. We witnessed gutting behavior in >95% of the *P. dominula* processing of kills of third and fourth instars in gardens. Similarly-excised digestive tracts left on milkweed leaves where sentinel third and fourth instars had been removed strongly implicates paper wasps, especially *P. dominula*, as the most likely factor accounting for the greater mortality of monarchs in urban gardens compared to rural sites. While it is possible that securing the sentinel larvae to the milkweeds in that trial may have overestimated predation by preventing larval escape (e.g., by dropping from the plant), it is unlikely to have had a major influence because in our direct observations, the majority (73%) of encounters between *P. dominula* and non-secured monarch larvae of that size resulted in predation. Chinese mantids, *Tenodera sinensis*, the only other invertebrate predator reported to gut larvae before consuming them^41^, were not observed feeding on monarchs in our gardens.

Butterfly “hibernation boxes” are typically made of wood with vertical slits intended for entry and bark lining the inside wall. Gardeners may add such structures to pollinator habitat with the intent of providing shelter for butterflies that overwinter as adults, or for ornamental interest^26,42^. Although there is little or no evidence that butterflies use such boxes, they are promoted in some gardening blogs and extension publications [e.g.^43^]. As shown herein, however, such boxes are good nesting sites for *P. dominula*. Their presence in urban butterfly gardens is likely to increase predation of the larvae those gardens are meant benefit. Similar wooden structures such as bird houses may also provide attractive nesting sites for *Polistes* wasps (authors’ observations).

Although our study was done in one metropolitan area, *P. dominula* is likely to impact monarchs wherever the two species’ distributions overlap. Indeed, we found online anecdotal accounts of *P. dominula* preying on monarchs in urban settings throughout the butterfly’s breeding range [e.g.^44^]. Although our exposing multiple sentinel larvae per plant may have overestimated typical field predation rates by evoking wasps’ functional response^45^, egg-loading tends to be greater in urban gardens compared to natural stands^16,17^ so it is common to find multiple larvae on a given milkweed plant^18^. Our trials were in mid-summer when *P. dominula* colonies had many workers, so they might have less impact on monarchs early in the growing season. The wasps can be managed by limiting access to preferred nest sites (e.g., repairing holes in walls, soffits and eaves; screening vents and louvers), treating exposed nests with a wasp and hornet spray, or applying insecticidal dust to cavities with nests^46^. Managing *P. dominula* may be necessary to prevent urban milkweed gardens from becoming ecological traps.

## Conclusion and Implications

Metropolitan areas provide a substantial canvas for monarch habitat restoration^26^ and their contribution may be essential to meet existing goals to increase milkweed abundance by 1.8 billion stems to support monarch butterflies^12^. Although numerous programs encourage urban and suburban citizens to plant gardens with milkweeds^13,14^, the assumption that such efforts will help reverse declining monarch abundance caused by habitat loss is largely untested. Urban gardens, which generally resemble small habitat patches, may be prone to repeated depredation in areas with high densities of *P. dominula* and other native paper wasps. Indeed, there is evidence that urban gardens may act as ecological traps for certain butterfly species (e.g., the pipevine swallowtail, *Battus philenor*) by attracting adults away from better quality habitat^47^.

Several previous authors cautioned that compared to natural habitat, urban milkweed gardens might expose monarchs to increased risk from pesticide exposure, disease, parasitism or predation^19,20,48^. Studies to date, however, are equivocal, some finding no difference in the overall low survival of monarch eggs and larvae between urban residential or natural sites^16,20^, and another suggesting higher mortality in gardens^48^. None of those papers identified particular predators contributing to larval attrition. Our study highlights *P. dominula* as an under-recognized threat to monarch larvae in urban gardens. The probable impact of this wasp should be considered in estimates of the current and potential contribution of milkweed in urban areas to monarch conservation, and in recommendations about where best to focus future restoration efforts.

## Methods

### Assessing *P. dominula* prevalence in urban gardens

Sixteen pre-existing urban pollinator gardens at residences, campuses, and businesses within the Lexington, Kentucky city limits were monitored for presence of foraging paper wasps. Observations took place throughout July 2019, on afternoons (1200–1700 h) of clear warm (>25 °C) days. Each garden was visited once by two independent observers who observed different portions of the garden for 30 min, recording numbers of independent wasp visits. Wasps exhibiting predatory searching behavior were counted; those nectaring at flowers were not. Observers stood on the perimeter of the gardens and tracked wasps from the time they entered the garden until they left. Each garden had unique features, but they were of similar size (mean 64.8 m^2^; range 42.8–80.3 m^2^), close to buildings, and contained a mixture of milkweeds and other flowering plants.

### *Polistes dominula* encounters with monarch larvae in gardens

We recorded outcomes of 120 encounters (30 each for second to fifth instars) between wild *P. dominula* foragers and monarch larvae feeding on mature swamp milkweed (*Asclepias incarnata*) in urban gardens. The milkweeds were grown from 2-yr old rootstock in a soil/bark mix in 5.7 liter pots and about 90 cm tall when used. Observations took place from 7–31 July at three pre-existing urban pollinator gardens, two of them (>300 m apart) on the University of Kentucky Lexington campus and the third at a residence about 3 km away. All gardens contained a similar mix of flowering nectar and butterfly host-plants; e.g., milkweeds (*Asclepias* spp.), spicebush (*Lindera benzoin*), asters (*Aster* spp.), cone flowers (*Echinacea* spp.) and others. Before each observation period, 10 monarch larvae (generally a mix of two successive instars, as available) were placed on separate leaves of an undamaged swamp milkweed, distributed throughout the plant, and allowed to establish for about 1 h. The plant was then placed in a garden and watched continuously for 90 min. All observations were on clear warm (>25 °C) sunny days between 1100–1700 h. Monarch larvae taken during a given observation period were not replaced. Fresh plants and larvae were used for each observation period.

### Predation on monarch larvae in urban and rural settings

Cohorts of ten monarch larvae (five each of third and fourth instars) were placed on each of twenty mature swamp milkweeds as above, and secured in place by inserting a fine (#00 insect pin) through the anal prolegs and leaf into a bit of cork on the opposite side which allowed them to feed but prevented their escape or loss from causes other than predation. On each plant, five larvae were pinned to the abaxial side of leaves and the remainder to adaxial surfaces. As a check for possible escapes, 30 larvae were similarly affixed to plants in the greenhouse, where 100% were still in place after 8 h.

On 4–5 August 2019, the aforementioned plants with sentinel larvae were placed in 10 urban gardens, and at 10 rural sites, left in the field for 8 h (1100–1900 h), and then inspected for signs of predation. Rural sites (mostly in nature parks and farm edges) were open meadow with pasture grasses and wild flowering plants, including milkweed, bordered by woodlots, whereas garden sites were all within the Lexington city limits. We used satellite images and the Measure Tool feature of Google Earth Pro geospatial software (Microsoft, Palo Alto CA) to measure distance from where each plant with larvae was placed to the nearest structure.

### Wasp exploitation of butterfly boxes in pollinator gardens

We observed *P. dominula* entering and exiting butterfly “hibernation boxes” that a student organization had placed in six, widely-spaced pollinator gardens on the University of Kentucky Campus (Fig. 4). To verify extent of colonization by paper wasps, we opened the 22 boxes in October 2019 to verify if they had been occupied, and by what species. Failed nests (<10 cells) were not counted. Wasps were still present on nests during the survey.

### Statistical analyses

Numbers of foragers of different *Polistes* spp. observed in urban gardens, relative proportions of monarch instars killed during or escaping encounters with *P. dominula*, and predation on sentinel larvae in urban gardens versus rural settings were compared by one-way analysis of variance, χ^2^ test for independence, and two-sample t-tests, respectively, using Statistix 10 (Analytical Software, Tallahassee, FL).

## Data Availability

All data are presented as totals or means ± SE in the main text. Raw data are available from the authors upon request.
